# Experimental Analyses of the Major Parameters Affecting the Intensity of Outbursts of Coal and Gas

**DOI:** 10.1155/2014/185608

**Published:** 2014-08-04

**Authors:** W. Nie, S. J. Peng, J. Xu, L. R. Liu, G. Wang, J. B. Geng

**Affiliations:** ^1^State Key Laboratory of Coal Mine Disaster Dynamics and Control, Chongqing University, Chongqing 400044, China; ^2^Key Laboratory of Ministry of Education for Mine Disaster Prevention and Control, Shandong University of Science and Technology, Qingdao 266510, China

## Abstract

With an increase in mining depth and production, the intensity and frequency of outburst of coal and gas have a tendency to increase. Estimating the intensity of outbursts of coal and gas plays an important role because of its relation with the risk value. In this paper, we described the semiquantitative relations between major parameters and intensity of outburst based on physical experiments. The results showed increment of geostress simulated by horizontal load (from 1.4, 2.4, 3.2, to 3.4 MPa) or vertical load (from 2, 3, 3.6, to 4 MPa) improved the relative intensity rate (3.763–7.403% and 1.273–7.99%); the increment of porosity (from 1.57, 2.51, 3, to 3.6%) improved the relative intensity rate from 3.8 to 13.8%; the increment of gas pressure (from 0, 0.5, 0.65, 0.72, 1, to 1.5 Mpa) induced the relative intensity rate to decrease from 38.22 to 0%; the increment of water content (from 0, 2, 4, to 8%) caused the relative intensity rate to drop from 5.425 to 0.5%. Furthermore, sensitivity and range analysis evaluates coupled factors affecting the relative intensity. In addition, the distinction with initiation of outburst of coal and gas affected by these parameters is discussed by the relative threshold of gas content rate.

## 1. Introduction

An outburst of coal and gas is defined as the rapid release of a large quantity of gas in conjunction with the ejection of coal from the solid face. Previous studies have recognized that these major parameters affecting outburst of coal and gas include stress condition, gassiness of coal seams, geological structures, and mechanical and physical properties of coal based on the hypothesis of comprehensive factors [1–3]. However, these parameters can also affect each other; for example, coal permeability is affected by the stress level and the presence of joints, cleats, and fractures; the stress regime is influenced by mining depth; and coal strength may be affected by gas pressure and moisture content in coal [2]. Thus, a key challenge is to understand to what extent the major parameters are contributing to the intensity of coal and gas outburst. In past physical model incorporating coal briquettes is a very important tool to simulate the outbursts of coal and gas [4–8]. Recently, the effects of coal strength, reservoir pressure, geostress, pressure gradient, and gas composition on outbursts are qualitatively demonstrated by the experimental results [9–12]. Furthermore, the quantitative relations between these major parameters and intensity of coal and gas outburst still need to be concerned. In the study, we describe the semiquantitative relations between major parameters (gas pressure, horizontal and vertical load, porosity, and water content) and intensity of the outburst by physical experiments. The relative intensity rate of coal and gas outburst, as the increment of impact factors, as a main index, is investigated. Then the sensitivity and range analysis evaluates the major factors impact on the relative intensity considering the interactions in their coupled. Next, a conceptual governing equation for links between major factors and output is tentatively put forward. Finally, we discuss the difference with initiation of outburst of coal and gas affected by these parameters by a relative threshold of gas content rate.

## 2. Methods

### 2.1. Apparatus and Procedure

**Figure 1:**
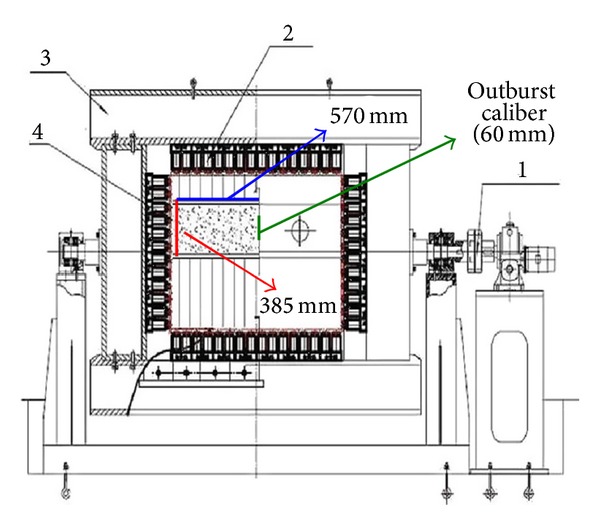
Structure of coal and gas outburst simulation test bed. (1) Revolving gear, (2) hydrostatic trigger, (3) bearing frame, and (4) coal body.

**Figure 2:**
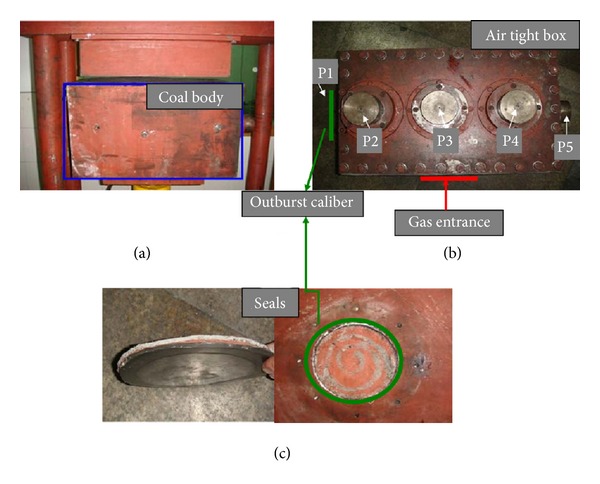
Key components. (a) Compression molded, (b) air tight box, and (c) seals.

**Figure 3:**
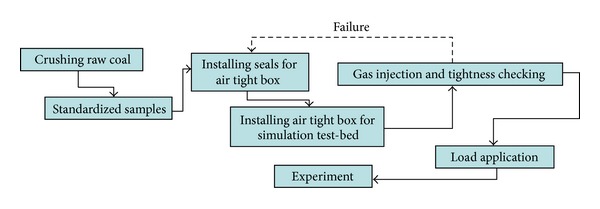
Flowchart of simulation test of coal and gas outburst.

**Figure 4:**
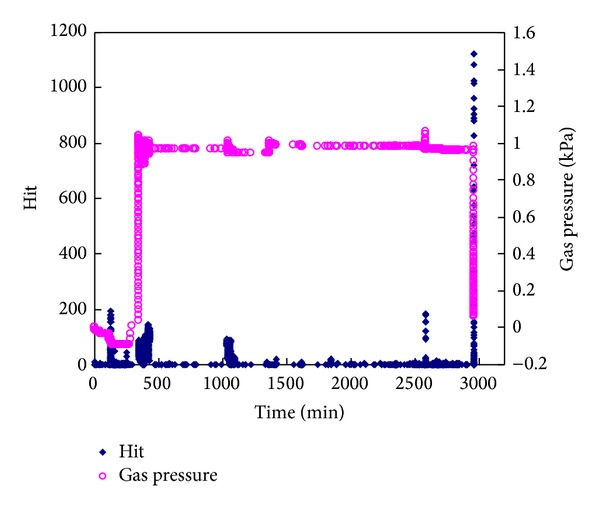
Monitoring data of gas pressure and Hit by acoustic emission during experiment (gas pressure −1 kPa and load pressure 3.6 MPa). 0–500 min: gas injection; ~2500 min: load application; ~3000 min: outburst.

Coal and gas outbursts with certain stress regimes, gas pressure, and materials property of samples are simulated by a coal and gas outburst simulation device consisting of fast-releasing components, a load bearing frame, electric self-controlled loading system, reversal unit, main frame bracket, and coal sample molding device as shown in Figure 1 [13]. Raw coal was crushed, screened, and compression-molded to produce standardized samples (briquette) under 4 MPa pressure (Figure 2(a)). Seals were installed between the mold and cover before lifting and pasting the mold sealing plate (Figure 2(c)). Then coal samples were filled in with gas through pressurization in an air tight box (Figure 2(b)). Before that an air-tightness test was required for the gas injection due to purity of experimental gas of 99.99%. Air extraction of vacuum needs to be operated about 2 hr before gas entering. After the gas entered the coal samples, full absorption of gas (about 48 hr) was the key step in the experiment. The gas pressure can be adjusted by a device during the gas absorption. After that as shown in Figure 2(b), horizontal load application is added firstly at outburst caliber (P1). The purpose is to prohibit the deformation of coal sample nearby outburst caliber. Then the vertical loads P2, P3, and P4 are applied orderly. At last, in order to keep the stress 3D stability another horizontal P5 is added facing the outburst caliber on the other side (for more details, see [14]). The outburst in coal and gas in the experiment is observed by opening the caliber fast. And the environmental temperature fluctuates between 18 and 20°C. The whole procedure is indicated in Figure 3.  Figure 4 indicates the monitoring data of gas pressure and Hit by acoustic emission during experiment.

### 2.2. Experimental Outlines

The purpose of experiments is to investigate what extent the major parameters affect the intensity of outburst of coal and gas. The investigated object is relative intensity which means the weight of coal outburst in the total experimental coal mass and and the relative intensity rate (RIR) which is defined as (1) meaning the variable rate of relative intensity along the impact factor increment:

(1) $$\begin{array}{c} {\text{RIR}}_{i+1}=\frac{\left(y_{i+1}-y_{i}\right)}{\left(x_{i+1}-x_{i}\right)}, \end{array}$$

where *i* = 1,2, 3,…, *y* is the investigated object, and *x* is the impact factor.

The whole experiments involved variables including vertical/horizontal load (geostress), gas pressure, moisture, and porosity generated by different particle size. In every experiment one variable is incremental and others are always fixed (Table 1). And the total test number is 23.

## 3. Results

**Figure 5:**
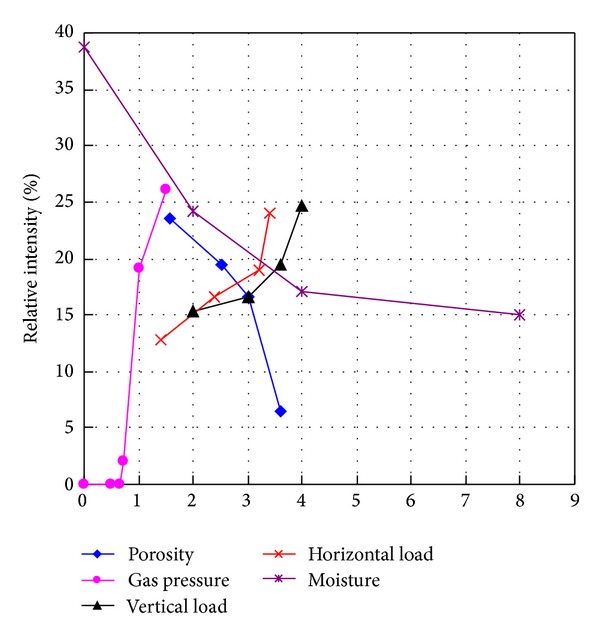
Factors increment versus relative intensity of outburst of coal and gas. Units in *X*-axis normalized are shown in Table 1.

**Figure 6:**
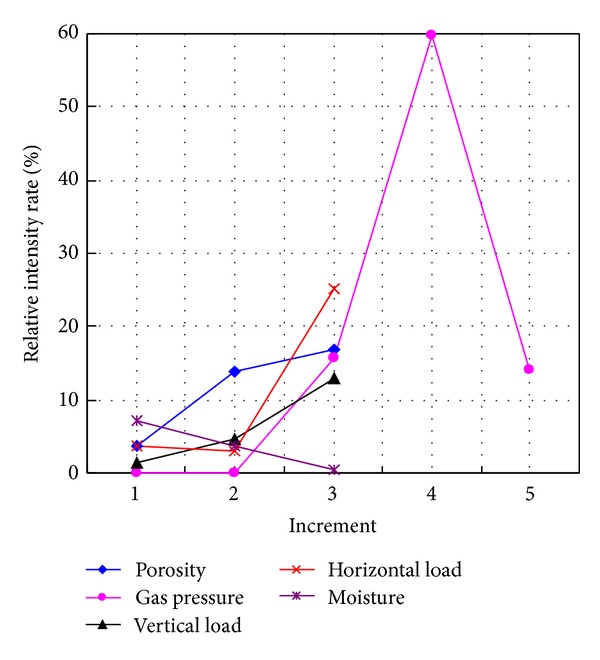
Factors increment versus relative intensity rate.

In this study, the relative intensities of outburst in coal and gas as factor increment are shown in Table 2. Their relationships are drawn in Figure 5, and Figure 6 demonstrates relative intensity rate (RIR) as increment of impact factors.

## 4. Discussions

### 4.1. Intensity of Outburst in Coal and Gas

The results show the vertical load and horizontal load, and gas pressures have a positive effect in increasing of the outburst intensity by contrasting the porosity and moisture with negative function. For the RIR, except the gas pressures and moisture with a decreased RIR, the other factors have an increase in RIR as factors increments. In this study, some factors are conceptually suggested into three types as follows: (1) direct factors such as geostress and material strength which usually determine the main energy of outburst failure, (2) indirect factors include gas pressure (produce gas pressure gradient to drive the outburst happen), water content, and porosity (both of which affect the coal strength), (3) catalysis factors such as gas adsorption and desorption which play a role to accelerate or inhibit the intensity of gas and coal outburst.

#### 4.1.1. The Effects of Moisture Content

As an essential parameter of physical properties, moisture content is investigated in this study. Microcosmically, it knows that gas phase pressure *P*_*g*_ and water phase pressure *P*_*w*_ are related by capillary pressure (*P*_*c*_), as expressed in the following equation:

(2) $$\begin{array}{c} P_{c}=P_{g}-P_{w}. \end{array}$$

Increase of *P*_*w*_ drops the *P*_*c*_; thus, the intensity of the outburst is low [15]. Macrocosmically, it also could be regarded that the high initial moisture content drops the strength of coal materials and furthermore reduces the outburst intensity. In other words if the confining pressure of rock is unchanged, the stronger material has more energy once the failure happens [16, 17] which means the intensity of the outburst is heavier. Another aspect, it basically regards that the water not only absorbs gas but also replaces gas to occupy some voids in the coal mass [18]. That means the number of gas adsorption and desorption reduces, so the RTR is dropped as an increment of moisture.

#### 4.1.2. The Effects of Porosity

The porosity as an influential factor also affects the strength of material in this study. The low porosity produces the stronger coal as what low moisture content does. Thus, they get the same result in Figure 5. RIR in Figure 6 shows gas adsorption induced by low porosity is not obvious, so the gas adsorption and desorption lead to reducing the RIR.

#### 4.1.3. The Effects of Gas Pressure

The gas pressure in coal links to gas pressure gradient. Paterson [19] took the general view that when gas is released from coal, there are body forces on the coal equal to the pressure gradients of the flowing gas. Litwiniszyn [20] mentioned the sudden creation of gas causes the skeleton of the medium to be destroyed. Thus, the intensity of outburst increases as gas pressure increment as shown in Figure 4. In our study, the gas pressure threshold to induce the outburst in coal and gas is 0.65–0.72 MPa. Thus, under gas pressure −0.65 MPa, the RI and RIR in both Figures 5 and 6 are zero. And once the gas pressure breaks through the interval, as shown in Figure 5 (step 2–step 4), the RIR increases swiftly. After that, while by the Langmuir adsorption [21] gas adsorption is enhanced under high gas pressure. It could be envisaged that RIR has an increased trend. However during the process of gas adsorption, the temperature dropped which conversely reduces the gas adsorption itself. In addition, crack initiation and growth with the function of free gas under certain geostress exist [22, 23] which is meant by the reduction of strength of material and intensity of gas outburst. Thus, the RIR seems to be dropped again. As a result, the RIR could depend on the balance between factors that increase or reduce gas adsorption. It should also be pointed out another explanation is that the homogeneous materials in the experiment have reduced RIR as increment of gas pressure while the heterogeneous coal has an almost constant intensity rate [24].

#### 4.1.4. The Effects of Vertical or Horizontal Load

A lot of researches showed the depth relates the geostress linearly which mainly concludes vertical and horizontal stress [25–27]. Thus, different geostress is simulated by changing the vertical or horizontal load in our experiment. If the confining pressure of rock increases, the material has more energy once the failure happens [28–30] as shown in Figure 5. However, another aspect, high geostress effect means the development rates of crack, joints, cleats, and fractures are faster [22, 23, 31]. The fracture development in the coal failure process will alter the permeability as well [32, 33], leading to a strong effect on the gas migration and distribution, furthermore inducing more damages. The process seems to consume the energy of the gas and coal outburst conversely. Unfortunately, some experiments showed under 4 Mpa earth pressure the development rate of cracks and fractures in coal mass is not obvious [34, 35]. Thus, the confining pressure induced by geostress still plays a main role in determining the intensity of outburst without considering the development of cracking and the RIR affected by gas adsorption is not changed obviously as shown in Figure 6.

#### 4.1.5. The Effects of Coupled Changes of Factors

In order to investigate the effects of coupled factors, we make use of orthogonal design to arrange the new experiments for testing sensitivity of moisture, geostress (vertical and horizontal stress keep changes in the meantime), porosity, and gas pressure to relative intensity in consideration of factor coupled [36]. Orthogonal design (Table 3) can use fewer experiments to replace the whole complete experiments (in the case 9 times tests equate 81 times tests).  Table 4 shows result of the range analysis that all major factors are affecting the relative intensity. For example, for moisture K1, K2, and K3 are cumulative values of factors of the first group test (no. 1~no. 3), second group test (no. 4~no. 6), and third group test (no. 7~no. 9), respectively. *R* equates maximum K1 reducing minimum K3, which means the range. Results of range analysis demonstrate *R*-Gas pressure is most significant factor compared to the weakest factor-*R*-porosity. And the *R*-geostress as well as *R*-moisture is at the middle level whose *R*-values are very close to each other. And the accurate quantitative relationship of these four factors could be calculated by differential analysis (see Table 5) [37].

Therefore, (3) could be considerd the method to estimate the relative intensity,

(3) RI=ω1×f1(x1)+ω2×f2(x2)+ω3×f3(x3)+ω4×f4(x4).

For rough calculation, the impact factors are related to the relative intensity linearly. The ordinary least squares (4) is used to calculate the coefficients based on multiple linear regression theory:

(4) $$\begin{array}{c} x^{*}=\underset{x}{\arg\;\min}{||b-\omega \times x_{i}||}_{F}^{2},\;\text{s}.\text{t}.\;\;x_{4}\geq 0.72, \end{array}$$

where *x*_*i*_ is the impact factor, *x*_4_ is the gas pressure (0.72 MPa is threshold of outburst), *b* is fitting coefficient, and *ω*_*i*_ is the weight of impact factor to RI.

The equation (5) in our study is to estimate the RI as follows:

(5) RI=−1.57044x1+3.337538x2+0.655278x3+6.599272x4,x4≥0.72,

where, *x*_1_,  *x*_2_,  *x*_3_, and *x*_4_ are moisture, geostress, porosity, and the gas pressure.

The Figure 8(a) indicates the relativity between model-equation (5) and measurement of outburst intensity; the residuals between model and measurement are shown in Figure 8(b).

#### 4.1.6. The Conceptual Governing Equation to Evaluate the Intensity of the Outburst

Under 4 MPa earth pressure, the development rate of cracks and fractures in coal mass is not obvious [34, 35]. Also, even under gas pressure −1.5 MPa, we still do not investigate the obvious fractures development by acoustic emission device as shown in Figure 4. Thus, in our study, relative independence could be considered among impact factors. Factors affecting relative intensity are in form of accumulated adding in (6) which is the conceptual governing equation. While (7) deciphers the impact factors related to the relative intensity rate,

(6) $$\begin{array}{c} \text{RI}=\overset{N}{\underset{i=1}{\sum }}\omega_{i}\times f_{i}\left(x_{i}\right), \end{array}$$

(7) $$\begin{array}{c} {\text{RIR}}_{i}=\frac{\partial \text{R}{\text{I}}_{i}}{\partial f_{i}}=\omega_{i}\times \frac{\partial f_{i}\left(x_{i}\right)}{\partial x_{i}}, \end{array}$$

where *i* = 1,2, 3,…, RI is the relative intensity, *x*_*i*_ is the impact factor, *ω*_*i*_ is the weight of the impact factor to RI, RIR_*i*_ is the relative intensity rate of impact factor *x*_*i*_, and ∂RI_*i*_/∂*f*_*i*_ is the partial derivative of RI to *x*_*i*_.

### 4.2. Initiation of Outburst of Coal and Gas

**Figure 7:**
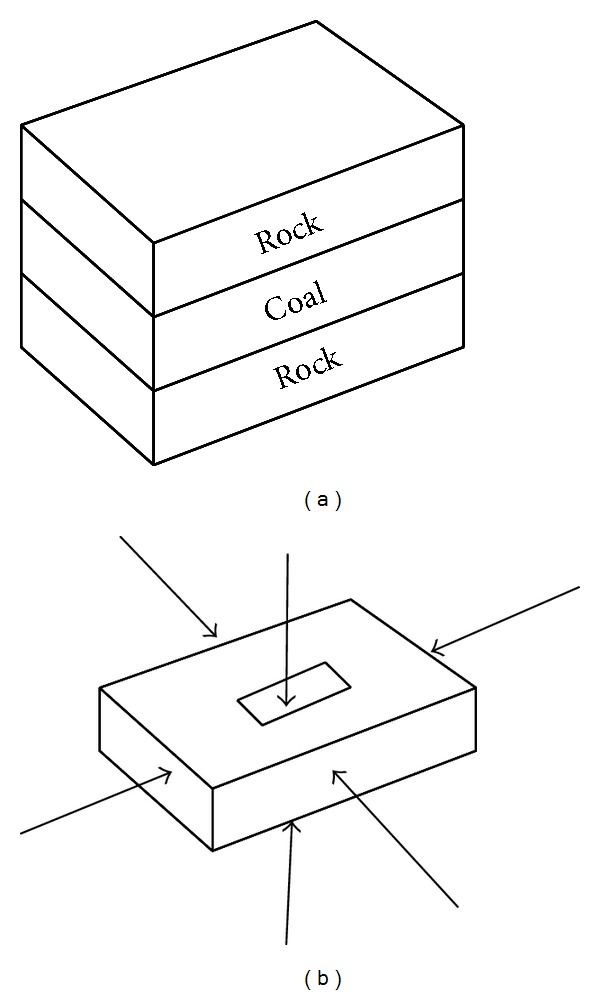
(a) Computational model and (b) model of a coal seam [15].

By contrast with parameters affecting the initiation of outburst of coal and gas, major parameters including the strength of coal, mining depth, and permeability contribute to the initiation of the outburst (Figure 9) [15]. As shown in Figure 10, the major parameters are plotted to relative threshold of gas content rate. And that computational and coal seam models are presented in Figures 7(a) and 7(b). The model results are in agreement with field observations [38, 39]. The gas content, which is usually as a threshold to judge the initiation of gas and coal outburst, means the total number of free gas and adsorption gas. And the relative threshold of gas content rate (RTR) is also investigated. Similarly, the RTR follows (1) which talks about the variable rate of threshold of gas content along the impact factor increment/decrement:

(8) $$\begin{array}{c} {\text{RTR}}_{j+1}=\frac{\left(y_{j+1}-y_{j}\right)}{\left(x_{j+1}-x_{j}\right)}, \end{array}$$

where *j* = 1,2, 3,…, *y* is the investigated object, and *x* is the impact factor. Here, the singular point is gas content as the threshold which affects the gas adsorption. As a result, catalysis factors affecting RIR are ignored. This paper simulated deep mining in real environment so that the strength of coal and mining depth (geostress) is inverse. As it is known both the strength and geostress have a proportional to the rock failure energy (intensity of the outburst). Thus, the threshold of gas content has no direct link with outburst intensity.

#### 4.2.1. The Effects of Strength in Coal

The uniaxial compressive strength, the most commonly used coal strength index, is selected to represent coal strength [15]. A lower uniaxial compressive strength value manifests more obvious vulnerable and more crack, joints, cleats, and fractures. So, less gas content in coal can induce outburst easily although the intensity of the outburst is not high surely. By contrast, stronger coal needs more gas content to break it. The coal with more crack, joints, cleats, and fractures is further damaged with the function of gas seepage [40, 41]. During the process of free gas transfers into adsorption gas, quite a bit coal is damaged by seepage force. Plus, recent research shows that gas adsorption reduces the uniaxial compressive strength and elastic modulus of coal [42, 43]. Thus, the RTR manifests more under weak coal and the threshold of the outburst is more sensitive to low strength in coal as shown in Figure 10.

#### 4.2.2. The effects of Permeability

Permeability in Figure 9 as a material property impacts the formation of gas gradient and gas adsorption rate. The low gas pressure gradient produced by high permeability leads to the high threshold of gas content for outburst because of quite a bit gas working for adsorption. While the low permeability is easier for development of high gas gradient, the threshold of gas content is lower. Another aspect, as the permeability increases, free gas transfers into adsorption gas more. And the free gas is considered the main reason to format the gas gradient. Thus, much more gas is required for maintaining the threshold of gas content for outburst. The adsorbed gas lowers the surface tension of coal and reduces the crack initiation threshold stress according to Griffith's failure criterion, decreasing the coal strength [44]. Thus, that is a kind of suppression to the high threshold of gas content by high permeability. So the RTR is elevated under low permeability. By contrast, for the coal with high permeability and already high gas adsorption, the increase of permeability does not reduce the free gas. As a result, the RTR is relatively low under high permeability as shown in Figure 10. In other words, the threshold of gas content is not sensitive to the high permeability.

#### 4.2.3. The Mining Depth

**Figure 8:**
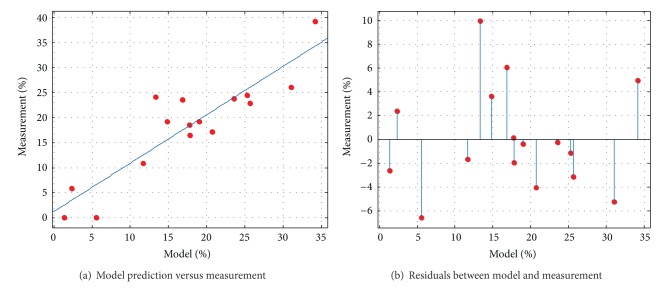
Evaluation of model predication (*R*-square: 0.8097 RMSE: 4.565).

**Figure 9:**
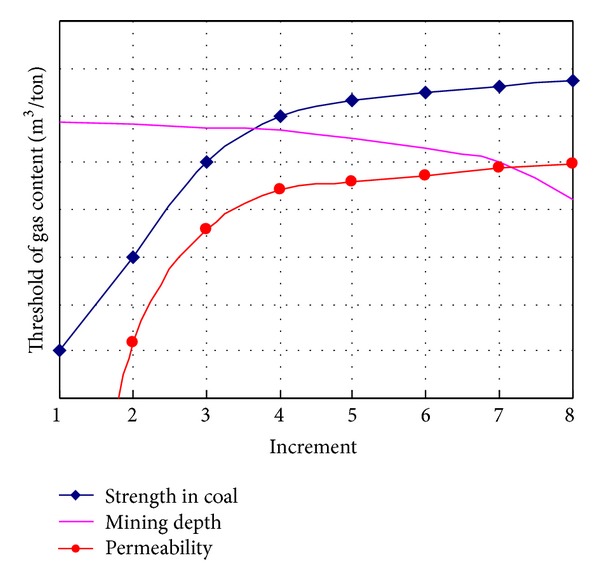
Major parameters affecting the initiation of outbursts of coal and gas [15].

**Figure 10:**
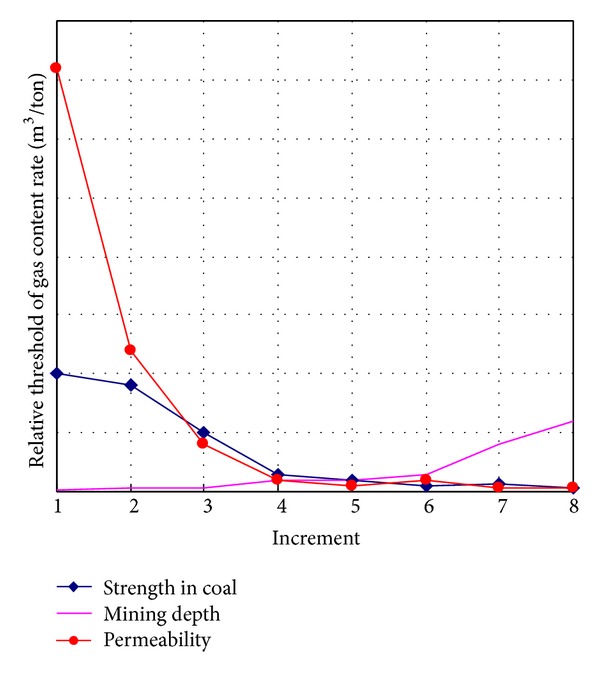
Major parameters versus relative threshold of gas content rate (permeability changed rate reduction faster than the changed rate of strength in coal while the mining depth changed rate increases as increment).

Mining depth as the parameter playing the geostress role in Figure 8 touches the threshold of gas content. High geostress means coal fractures are faster developed. And the fast development rate of crack and fracture is prone to outburst of gas and coal [39]. And in the meantime, the effective stress on coal increases. So the gas as induced factor does not need more. In addition, crack initiation and growth with the function of free gas under high geostress are exponent speed [22, 23]. Thus, its RTR is higher than that of low geostress as shown in Figure 9.

## 5. Conclusions

The parametric studies reported in this paper were carried out with a newly developed physical simulator to investigate the effects of some major factors contributing to the intensity of outburst of coal and gas. When the relative weight of an outburst in coal is used as an outburst index, the results from this study show that the outburst risk and the increasing risk rate vary as affecting factors increment or decrement. We also investigate the effects of coupled factors to the outburst intensity and put forward a conceptual governing equation to link them. In addition, the initiation of outburst under the changed strength in coal, mining depth, and permeability and the threshold rate is compared in the paper. Major parameters are influencing the intensity of outbursts link with the expected loss in case of accident. By contrast, the initiation of outburst of coal and gas plays a role of probability of the accident, of which we construct the risk of gas and coal outburst in mines [45]. Thus, in further quantitative relations between these major parameters and gas and coal outburst should be emphasized.

## Figures and Tables

**Table 1:** Experiments program.

Level	Vertical load (MPa)	Horizontal load (MPa)	Moisture (%)	Gas pressure (MPa)	Porosity (%)
1	2	1.4	0	0	1.57
2	3	2.4	2	0.5	2.51
3	3.6	3.2	4	0.65	3
4	4	3.4	8	0.72	3.6
5	—	—	—	1	—
6	—	—	—	1.5	—

Remarks: the fixed values are 3 MPa (vertical load), 2.4 MPa (horizontal load), 4% (moisture), 1 MPa (gas pressure), and 2.51% (porosity).

**Table 2:** Relative intensity of outburst in coal and gas.

Relative intensity of outburst (%)					
Level	Vertical load	Horizontal load	Moisture	Gas pressure	Porosity
1	15.345	12.855	38.7	0	23.57
2	16.618	16.618	24.21	0	19.43
3	19.4	19	17	0	16.6
4	24.608	24.021	15	2.1	6.47
5	—	—	—	19.11	—
6	—	—	—	26.11	—

**Table 3:** Coupled factors and relative intensity of outburst in coal and gas.

Factors					Results of experiments
Test number	Moisture (%)	Geostress (MPa)	Porosity (%)	Gas pressure (MPa)	Relative intensity (%)
1	0	3.4	3.1	3	39.2
2	3.6	1.9	3.3	1.34	10.9
3	2.6	1.8	2.3	1.5	24.1
4	2.4	2	3.6	1.9	16.5
5	3.6	1.3	2.4	3.1	17.2
6	2.8	3.3	0.5	1.5	23.57
7	3.4	3.4	1.7	1.8	19.2
8	2.6	4	3.3	2.1	24.5
9	4	4	3.1	2.2	23.8
10	4.8	3	2.8	1.6	19.23
11	3.6	4	3.3	2.4	22.9
12	8	3.8	2.2	0.6	0
13	2.8	4	3.6	3.6	26
14	7.2	1	3.6	1.2	5.87
15	7.2	2.1	1.4	3.2	18.5
16	0	0.6	0.6	0	0

Remarks: moisture: level 1 (0–2.7), level 2 (2.7–5.4), and level 3 (5.4–8); Geostress: level 1 (0–1.33), level 2 (1.33–2.66), and level 3 (2.66–4); Porosity: level 1 (0–1.2), (1.2–2.4), level 3 (2.4–3.6); gas pressure: level 1 (0–1.33), level 2 (1.33–2.67), and level 3 (2.67–4).

**Table 4:** Orthogonal design and result.

Factors levels					Results of experiments
Test number	Moisture	Geostress	Porosity	Gas pressure	Relative intensity (%)
1	1	1	1	1	0
2	1	2	2	2	24.1
3	1	3	3	3	39.2
4	2	1	2	3	17.2
5	2	2	3	1	10.9
6	2	3	1	2	23.57
7	3	1	3	2	5.87
8	3	2	1	3	18.5
9	3	3	2	1	0

**Table 5:** Range analysis and the result.

Level	Moisture	Geostress	Porosity	Gas pressure
K1	21.1	7.69	14.023	3.63
K2	17.223	17.83	13.77	17.85
K3	8.123	20.923	18.66	24.97
*R*	12.98	13.23	4.887	21.34
