# Erratum to “Experimental Analyses of the Major Parameters Affecting the Intensity of Outbursts of Coal and Gas”

**DOI:** 10.1155/2014/461540

**Published:** 2014-12-08

**Authors:** W. Nie, S. J. Peng, J. Xu, L. R. Liu, G. Wang, J. B. Geng

**Affiliations:** ^1^State Key Laboratory of Coal Mine Disaster Dynamics and Control, Chongqing University, Chongqing 400044, China; ^2^Key Laboratory of Ministry of Education for Mine Disaster Prevention and Control, Shandong University of Science and Technology, Qingdao 266510, China

Here, we correct the errors made in Table 3 and equation (5) in the paper titled “Experimental Analyses of the Major Parameters Affecting the Intensity of Outbursts of Coal and Gas.” The purpose of this paper is to correct both the data input and the mathematical errors in Table 3 and equation (5).

The data input (Gas pressure (Mpa)) in Column 5, Table 3, should be as in the table mentioned in this paper.

The coefficients in equation (5) should be changed to

(5) RI=−1.5874x1+2.866x2−0.3791x3+19.567x4,x4≥0.72.

## Figures and Tables

**Table 3:** Coupled factors and relative intensity of outburst in coal and gas.

Factors					Results of experiments
Test number	Moisture (%)	Geostress (MPa)	Porosity (%)	Gas pressure (MPa)	Relative intensity (%)
1	0	3.4	3.1	1.5	39.2
2	3.6	1.9	3.3	0.75	10.9
3	2.6	1.8	2.3	1	24.1
4	2.4	2	3.6	0.75	16.5
5	3.6	1.3	2.4	1.5	17.2
6	2.8	3.3	0.5	0.75	23.57
7	3.4	3.4	1.7	1	19.2
8	2.6	4	3.3	1	24.5
9	4	4	3.1	1	23.8
10	4.8	3	2.8	0.75	19.23
11	3.6	4	3.3	1	22.9
12	8	3.8	2.2	0.5	0
13	2.8	4	3.6	1.5	26
14	7.2	1	3.6	1	5.87
15	7.2	2.1	1.4	1	18.5
16	0	0.6	0.6	0	0

Remarks: moisture: level 1 (0–2.7), level 2 (2.7–5.4), and level 3 (5.4–8); geostress: level 1 (0–1.33), level 2 (1.33–2.66), and level 3 (2.66–4); porosity: level 1 (0–1.2), (1.2–2.4), and level 3 (2.4–3.6); gas pressure: level 1 (0–0.5), level 2 (0.5–1), and level 3 (1–1.5).

